# A Scalable Digital Light Processing 3D Printing Method

**DOI:** 10.3390/mi15111298

**Published:** 2024-10-25

**Authors:** Junjie Huang, Jiangkun Cai, Chenhao Huangfu, Shikai Li, Guoqiang Chen, Hao Yun, Junfeng Xiao

**Affiliations:** 1 School of Mechanical and Power Engineering, Henan Polytechnic University, Jiaozuo 454003, China; 2 Shenzhen Institute for Advanced Study, University of Electronic Science and Technology of China, Shenzhen 518000, China; 3 ShenSi Shape (Shenzhen) Technology Co., Ltd., Shenzhen 518000, China

**Keywords:** 3D printing resolution, 3D printer, digital light processing, scalable printing, shape accuracy

## Abstract

The 3D printing method based on digital light processing (DLP) technology can transform liquid resin materials into complex 3D models. However, due to the limitations of digital micromirror device (DMD) specifications, the normal DLP 3D printing method (NDPM) cannot simultaneously process large-size and small-feature parts. Therefore, a scalable DLP 3D printing method (SDPM) was proposed. Different printing resolutions for a part were designed by changing the distance between the projector and the molding liquid level. A scalable DLP printer was built to realize the printing resolution requirements at different sizes. A series of experiments were performed. Firstly, the orthogonal experimental method was used, and the minimum and maximum projection distances were obtained as 20.5 cm and 30.5 cm, respectively. Accordingly, the layer thickness, exposure time, and waiting leveling time were 0.08 mm, 3 s, and 6 s and 0.08 mm, 7 s, and 10 s. Secondly, single-layer column feature printing was finished, which was shown to have two minimum printing resolutions of 101 μm and 157 μm at a projection distance of 20.5 cm and 30.5 cm. Thirdly, a shape accuracy test was conducted by using the SDPM. Compared with the NDPM, the shape accuracy of the small-feature round, diamond, and square parts was improved by 49%, 42%, and 2%, respectively. This study verified that the SDPM can build models with features demonstrating high local shape accuracy.

## 1. Introduction

Three-dimensional printing, also known as the additive manufacturing technology, differs from traditional processing methods, performing solid manufacturing by stacking materials layer by layer. Various 3D printing techniques, such as fused deposition modeling [1,2], direct ink writing [3,4], selective laser melting [5,6], and stereolithography [7,8], have been widely used in the automotive [9], aerospace [10,11], bioprinting [12], and construction [13] fields, among others. Stereolithography is the typical 3D printing technique that allows complex parts to be manufactured directly from liquid photosensitive resins. Digital light processing (DLP) based on 3D printing technology is a type of stereolithography. The DLP 3D printing method selectively cures liquid resin layer by layer based on the pattern projected onto the photosensitive resin surface, which is different from other types of 3D printing methods of stereolithography that use dots or lines to form a two-dimensional layer. Therefore, DLP 3D printing technology usually possesses high efficiency in processing large-volume and complex 3D model structures [14], and it has the advantages of rapid speed, high resolution, and good surface quality [15]. Currently, DLP 3D printing technology is widely used in microfluidics [16,17], metamaterials [18], biomedicine [19], and 4D printing [20].

**Figure 1:**
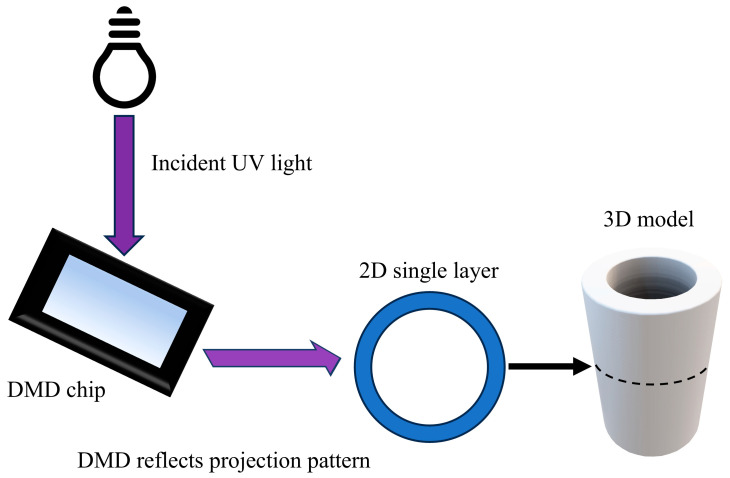
DLP 3D printing process.

Despite the above-mentioned advantages, however, DLP printing also suffers from some limitations concerning accuracy and size. Figure 1 illustrates the basic principle of the DLP 3D printing method. The DMD chip, which consists of millions of micromirrors, is the key component for the DLP projection system. Each micromirror can be independently controlled to rotate. The digital micromirrors on the DMD chip selectively reflect incident UV light to form a projection pattern and cure resin layer by layer so that the model can be manufactured. In the projection system, the number of micromirrors of the DMD chip determines the resolution of the projection pattern, and the size of the projection format depends on the lens specifications. In addition, each micromirror represents a pixel, so increasing the print format will lead to low accuracy. Therefore, it is worth investigating how to balance size and accuracy requirements in the DLP 3D printing process.

Research on the printing size and accuracy of DLP 3D printing can be divided into two main types. One approach is to improve the accuracy but maintain the size. Specifically, Zhou et al. [21] proposed a subpixel shifting method, which improved the printing accuracy and resolution without sacrificing efficiency; Montgomery et al. [22] developed a pixel-level grayscale control method to create circular features from sharp pixels by using pixel-level grayscale control beyond the typical resolution of a given projector; and Zhou et al. [23] introduced a hybrid exposure concept. This consists of vector scanning and mask image projection subsystems, where the vector scanning system forms the boundary, and the mask projection system forms the inside area. The second approach is to maintain molding accuracy and expand molding size. He et al. [24] implemented a dynamic projection scanning method for large-part printing. This creates large parts with high resolution by moving the projector so that the projection image can be dynamically and continuously exposed on the resin surface. Xu et al. [25] presented a method that continuously moves the projector via rotation with a galvo mirror. This process is able to build 3D macroscale objects with microscale features. Wu et al. [26] designed a multi-projector DLP 3D printing scheme with energy homogenization. This effectively expands the printing size, but the scheme with multiple projectors increases the development costs. Yi et al. [27] first applied a Delta 3-DoF parallel mechanism to DLP 3D printing, where the forming platform with a parallel mechanism can be moved horizontally to print large models. However, due to the limitations of the mechanical structure, its motion control is complicated. Furthermore, there have been other attempts to solve the conflict between molding size and accuracy. Wang et al. [28] mounted a set of optical lenses at the front of the projector and made changes to the dimensions of the projection pattern by moving the convex lens to achieve different printing sizes and resolutions. Moreover, Wang et al. [29] printed the large-size objects and high-accuracy features by using double projectors with the exact resolution.

In the actual DLP 3D printing, it is not necessary for all the features within a given model to attain the same degree of accuracy. In light of this, a scalable DLP 3D printing method (SDPM) is proposed. In this study, a self-designed scalable printer is employed. The fabrication of the large-size part is initiated, followed by the movement of the projector to adjust the projection distance, thus enabling the fabrication of the small-feature part. The print format and the print pixel size of the different parts in one model are varied, thus realizing the requirements of printing accuracy under different molding sizes.

## 2. System and Methods

### 2.1. Printing Principle

**Figure 2:**
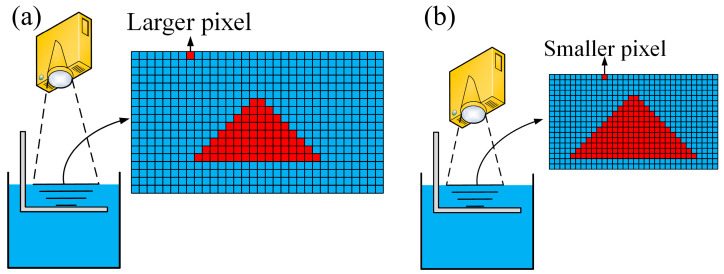
Schematic diagram of scalable DLP molding: (**a**) large-size printing; (**b**) small-feature printing.

Figure 2 illustrates the molding principle of the SDPM when the large-size and the small-feature parts are printed. Each grid in the molding format represents a projection pixel. It can be seen from Figure 2 that the difference between the dimensions of large-size and small-feature molding formats is obvious, even if the number of pixels is the same. The red triangle pattern exhibits identical molding sizes in both printing formats. However, the small-feature printing format exhibits a higher pixel density and a better fit to the actual model. The projector, as the core device of the DLP 3D printing system, determines the size of the molding format and molding pixel, which is defined by the projection ratio:

*T* = *D*/*W* (1)

where *T* is the projection ratio, *D* is the projection distance, and *W* is the width of the molding format on the liquid resin surface. The smaller the *D*, the smaller the *W*, and the smaller the pixel size obtained, which results in the higher accuracy of the projection pattern. For a model with a large overall dimension and several small features distributed over it, the projection distance can increase to obtain a larger molding format for printing the large-size part. Subsequently, the projector moves to reduce the projection distance to obtain a smaller size of molding pixels for small-feature printing. The parameters of the DLP projector selected for this paper are listed in Table 1.

According to the structural characteristic of the model, the projector is moved to change the projection distance so that it can be flexibly adapted to the printing size and accuracy requirements for a model.

### 2.2. Printing Device and Procedure

**Figure 3:**
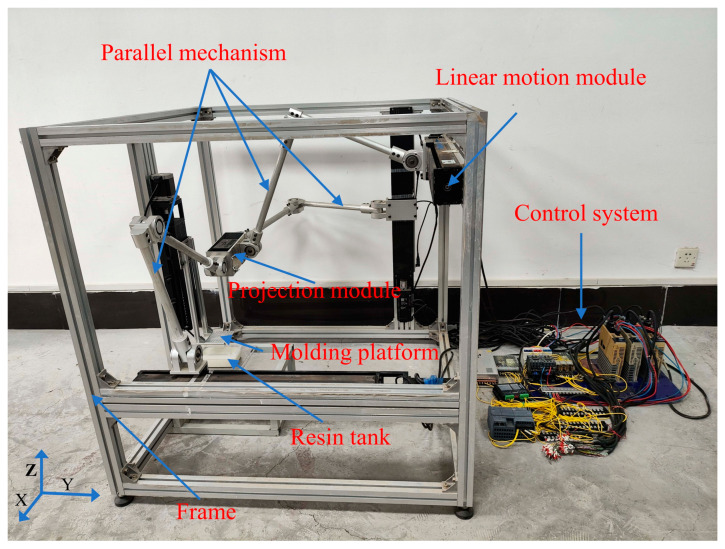
Scalable DLP printer.

To realize the SDPM, a self-developed scalable DLP printer is used [30], as shown in Figure 3. The printing device consists of a frame, a parallel mechanism, three linear motion modules, a projection module (projector with 405 nm light source), a resin tank, and a molding platform. The parallel mechanism consists of three branch chains (PRRR), each of which contains one prismatic pair (P) and three rotating pairs (R). One end of the branch chain is connected to a linear motion module, and the other end is connected to the projection module. The three linear motion modules are arranged along the X/Y/Z axial direction, and they are orthogonally set on the frame. The projector is mounted inside the projection module and uses a top-down approach to expose images. The resin tank is placed below the projection module, and the linear motion module drives the molding platform individually.

During the printing process, the parallel mechanism drives the projection module to transform position in 3D space, so that the projection distance can be adjusted by the movement of the projection module.

**Figure 4:**
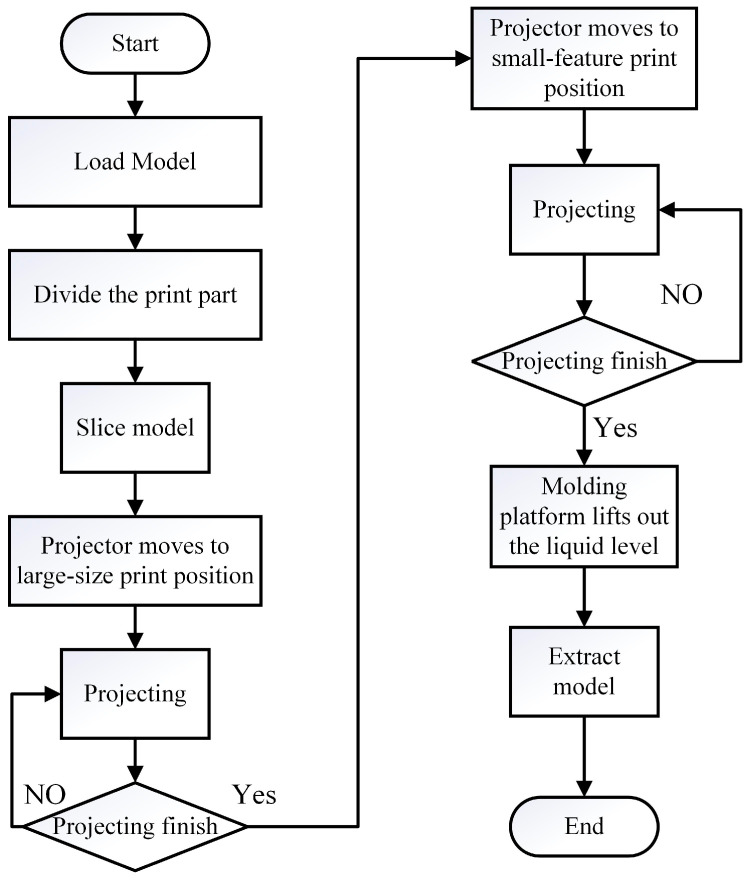
Printing procedure.

For the model with the large-size part and the small features, different accuracy is required for completion. Accordingly, the SDPM printing procedure is as follows: first, the 3D model is divided into a large-size part and several small-feature parts according to the characteristic of the model, and the projection images are generated; second, the projector is moved to the origin position to fabricate the large-size part. Then, the parallel mechanism moves the projection module, which makes the projector shift to the corresponding position of small features. Finally, the small features are built. The specific printing procedure is illustrated in Figure 4.

## 3. Experiments and Discussion

In this section, three groups of tests, including the determination of printing parameters, printing resolution, and shape accuracy, are designed and conducted to evaluate the scalable DLP 3D printer, and then the scalable printing is performed. To ensure the flatness of the liquid surface for each printed layer, the distance that the molding platform moves down must be greater than the thickness of each layer at a time, followed by lifting and waiting for the resin to level out. This approach allows the resin to cover the molding platform quickly and avoids uneven resin coverage due to thin layer thickness. The properties of the photosensitive resin used are shown in Table 2. The resin viscosity is low and the liquid flow rate is fast, which is conducive to the stability of the liquid reflux. In addition, the cleaning method is water washing, which is easy to operate.

### 3.1. Determination of Printing Parameters

In the scalable printer, the printing effect and capability depend on the layer thickness, exposure time, and waiting leveling time. An orthogonal experimental method is used for the above printing parameters. The three factors set for the test are the layer thickness (A), exposure time (B), and waiting leveling time (C). The minimum projection distance of the projector is set as 20.5 cm. The orthogonal test is conducted on the printing parameters at a projection distance of 20.5 cm, and three levels are considered for each factor. The values of each factor are initially selected as shown in Table 3.

Nine sets of tests are presented using the L_9_(3^4^) orthogonal table. The test sample is a rectangular block of 15 mm (X) × 20 mm (Y) × 3 mm (Z). A vernier caliper (Harbin Measuring & Cutting Tool Group CO., LTD, Harbin, China) is used to measure the dimensions of the printed samples in the X, Y, and Z directions, and five measurements are taken for each sample to obtain the average value. Using the relative size error (RSE) to represent the dimensional accuracy of the test sample,

*RSE* = |*A*0 − *A*1|/*A*1 × 100%, (2)

where *A*0 is the measured value and *A*1 is the theoretical value. The *RSE* of each direction is denoted by ΔX, ΔY, and ΔZ, respectively. The results of nine sets of orthogonal tests are shown in Table 4.

The measured data demonstrate that the sample has the most extensive *RSE* range in the Z direction (1.9%), so the *RSE* of the Z direction is selected as the representation for dimensional accuracy. The range analysis results are given in Table 5.

**Figure 5:**
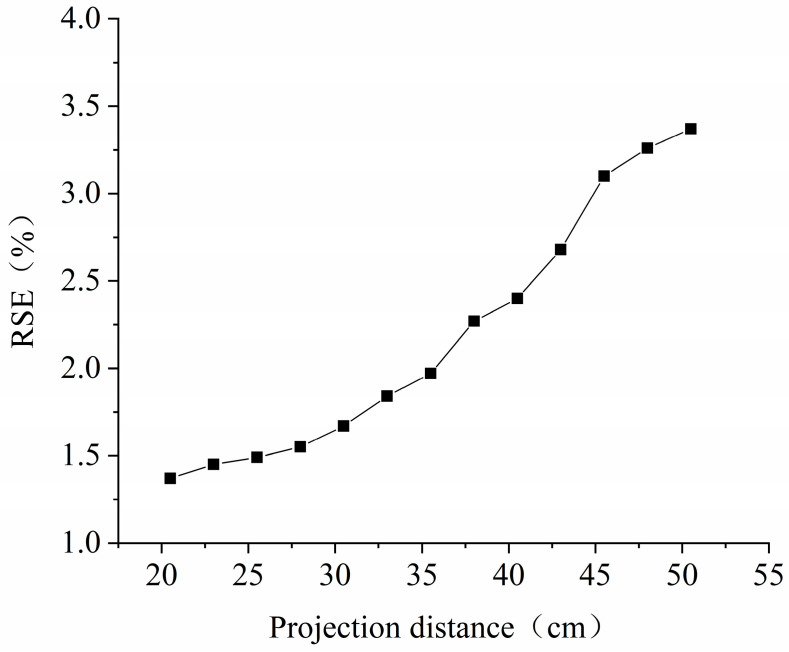
Plot of dimensional accuracy versus projection distance.

The parameter with the higher range R has a more significant influence on dimensional accuracy. Analyzed from the range results, the three parameters affecting the printing effect are A > B > C. They are in the order of layer thickness, exposure time, and waiting leveling time. The smaller the value of K, the smaller the RSE, and the optimal combination is A_1_B_2_C_1_. Therefore, the printing parameters are a layer thickness of 0.08 mm, exposure time of 3 s, and waiting leveling time of 6 s. Considering that the printing parameters will change with the projection distance, orthogonal tests were carried out at intervals of 2.5 cm to determine the printing parameters and to measure the dimensional accuracy of the samples, as shown in Figure 5.

As can be seen from Figure 5, the RSE of the printed model is not linearly related to the projection distance. As the projection distance increases, the RSE of the printed model will also increase, and the rate of increase will be accelerated. This is because the light intensity and uniformity are reduced as the molding format width increases in the constant light power. Enlarging the printing size by continuously increasing the projection distance is impossible. The increase in RSE is 0.3%, 0.73%, and 0.97% for the projection distance range of 20.5–30.5 cm, 30.5–40.5 cm, and 40.5–50.5 cm, respectively. In order to ensure the printing accuracy of the model as much as possible, the projection distance is set to vary within 20.5–30.5 cm, 20.5 cm is set to be the small-feature printing distance, and 30.5 cm is set to be the large-size printing distance. The parameters under a large-size printing distance are a layer thickness of 0.08 mm, exposure time of 7 s, and waiting leveling time of 10 s. The molding format sizes and pixel widths at the two distances are shown in Table 6.

### 3.2. Printing Resolution Test

**Figure 6:**
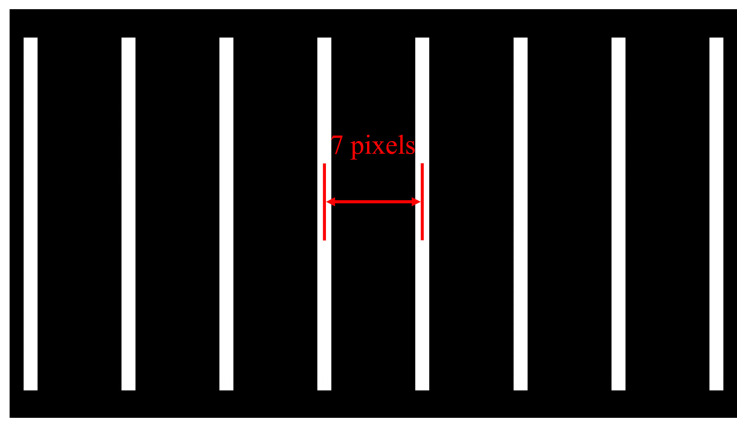
Column pixels pattern.

The single-layer printing resolution of the scalable printer determines the minimum feature size that the SDPM can achieve, which affects the printing accuracy of the model. A single-layer printing resolution test is designed to make the smallest features. The several pixels are arranged to form the column pattern as the projection image, as shown in Figure 6, and the distance between these columns is 7 pixels.

**Figure 7:**
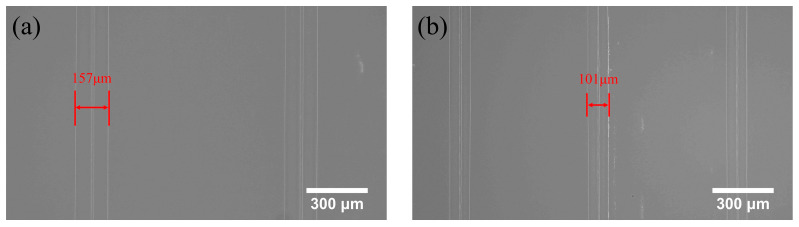
Minimum feature size: (**a**) 30.5 cm projection distance; (**b**) 20.5 cm projection distance.

The projection image is transferred from the computer to the projector for printing, and ten layers are first printed as the base layer. Then, the single-layer columns of the pixels are exposed and cured at the large-size projection distance of 30.5 cm and the small-feature projection distance of 20.5 cm. An industrial microscope BC3630 (Bocheng Electronics, Dongguan, China) was used to observe and measure the size of the cured structure, as shown in Figure 7. 

The results in Figure 7 show that the average width of the pixel at large-size and small-feature projection distances is 157 μm and 101 μm, representing the highest printing resolution. It can also be seen that the column pixel feature sizes of both large-size and small-feature printing distances are larger than their theoretical projection pixel sizes, with errors of 13.5 μm and 4.5 μm, respectively. The reason is that the photosensitive resin outside the boundary of the projection column pixel can be cured because of the light diffraction and chemical diffusion of DLP light, resulting in lower resolution. Thus, the minimum feature size of the actual printed column pixel is slightly larger than the theoretical one.

### 3.3. Shape Accuracy

**Figure 8:**
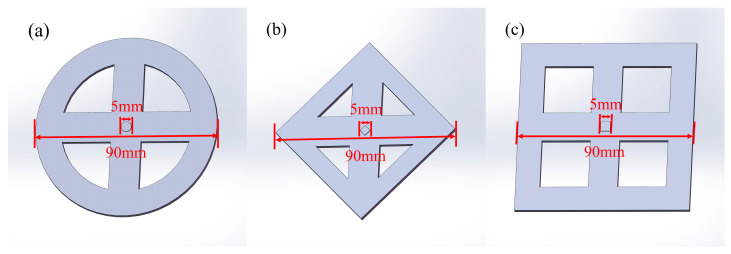
Shape accuracy test models: (**a**) double-round model; (**b**) double-diamond model; (**c**) double-square model.

The scalable printing method allows for fabricating features with higher shape accuracy because it has small-size pixels to fit the feature shape better. The models (a), (b), and (c) in Figure 8 are designed for the shape accuracy test. The three models are called double-round, double-diamond, and double-square. Each model consists of the external large-size and the central small-feature part. The large-size part is at the bottom of the model. The small-feature part is convex and located in the geometric center. The printing sizes are 5 mm for all small-feature parts and 90 mm for all large-size parts. The shapes of the small-feature parts are round, diamond, and square, characterized by circular arcs, bevels, and straight-line edges, respectively. The three edges are the essential elements that make up a variety of graphics. The effect of pixel size on the shape accuracy is representative.

**Figure 9:**
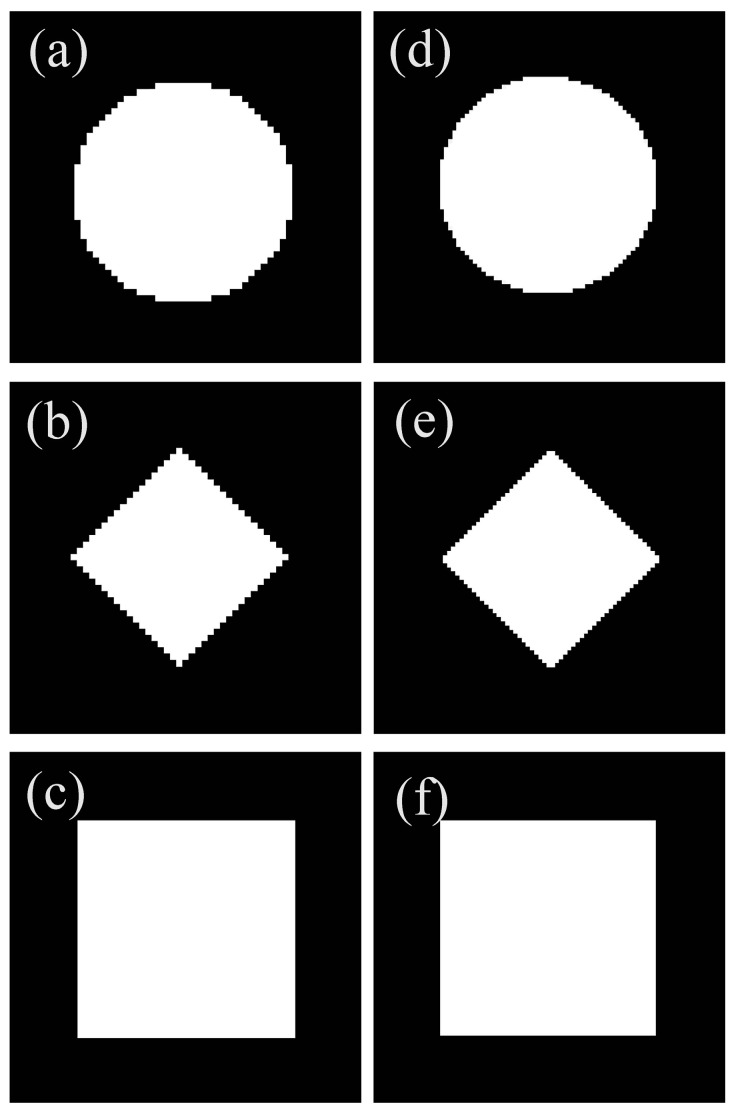
Projection images of small-feature parts: (**a**–**c**) projection images of small-feature parts for NDPM; (**d**–**f**) projection images of small-feature parts for SDPM.

The 2D digital patterns with the white cured area in the BMP format (Figure 9a–f) are the projection images of small-feature parts. As can be seen from the projection images, the projection images of square features for NDPM (seen in Figure 9c) and SDPM (seen in Figure 9f) have the same shape accuracy. However, the projection images of round and diamond features for NDPM (see Figure 9a,b) have a lower shape accuracy than those for SDPM (see Figure 9d,e). This is because the projection images of small-feature round and diamonds have circular arcs and bevels, respectively. Due to the square shape of the DMD micromirror, the shape of the square patterns can be fitted by pixel perfectly with big or small sizes. However, square pixels cannot entirely match the round and diamond patterns. Moreover, the accuracy will decrease with the increase in the pixel size.

**Figure 10:**
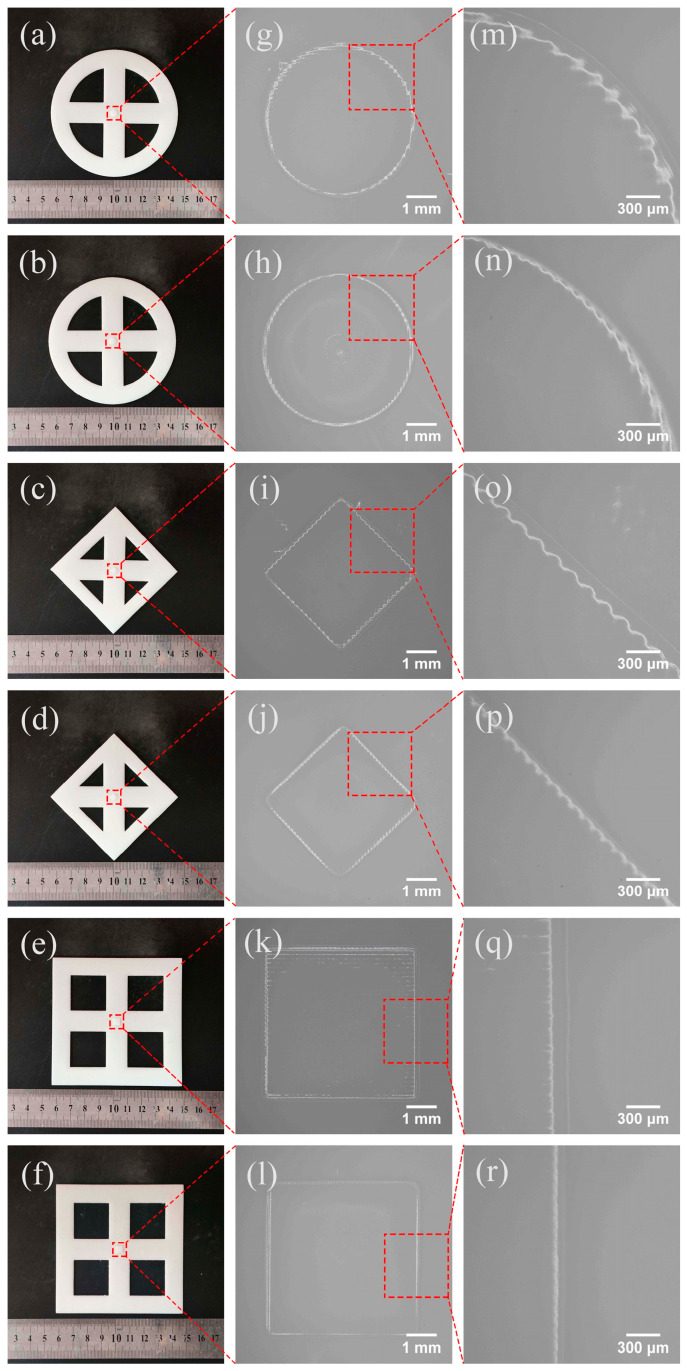
Shape accuracy test: (**a**,**c**,**e**) printed models of NDPM; (**b**,**d**,**f**) printed models of SDPM; (**g**–**l**) microscope shots of small-feature parts; (**m**–**r**) local edges of small-feature parts.

When printing using the SDPM, the scalable printer prints the large-size part first at a projection distance of 30.5 cm, followed by the small-feature part at 20.5 cm. Compared with the SDPM, both the large-size part and the small-feature part were printed at a projection distance of 30.5 cm when using the NDPM. The layer thickness is set to 0.08 mm, with 30 layers for large-size parts and 10 for small-feature parts. Each model is printed five times under NDPM and SDPM, respectively. Then, the small-feature parts are magnified and photographed by an industrial microscope BC3630 (Bocheng Electronics, Dongguan, China), as shown in Figure 10. Using the industrial microscope software’s (S-EYE_Setup-1.4.3.479-YW) circular and straight-line measurement tools, we can extract the circular and straight-line boundaries of the small-feature parts from Figure 10 and measure the shape accuracies. The measured results are shown in Table 7.

Figure 10 shows the models built using both NDPM and SDPM. The models show that obvious jagged edges occurred on both the round and diamond. This is attributed to the fact that the projection image consists of a series of discrete pixels, and each of the pixels is derived by a separate DMD micromirror. The DMD micromirror is square, and the projection image does not fit the original pattern perfectly, which causes the distortion of the image information and jagged edges. The jagged edges are reflected in the actual printed models. The edges of the models are not smooth. In addition, the edges of the small-feature parts are not strictly jagged, and the degree of jaggedness is softer than that of the theoretical edges. The reason is that the light intensity of each DMD pixel is not uniform, and its energy obeys a Gaussian distribution, where the light energy is attenuated from the center of the pixel to the edges. Therefore, the actual printed edge shape is unable to match the theoretical edge shape.

The accuracy measuring results show that the average roundness of the round decreases from 83 μm to 42 μm and the average straightness of the diamond reduced from 68.8 μm to 39.6 μm in NDPM and SDPM. Regarding the shape accuracy of the square, the average straightness of NDPM is 18.4 μm, which is close to that of SDPM (18 μm). The improvement of shape accuracy in the square feature is 2%, much smaller than that of round and diamond samples (49%, 42%). This is because the projection images of the square features for both NDPM and SDPM have the same shape accuracy, and the shape accuracies of the projection images of the round and diamond features for SDPM are higher than those for NDPM. The 2% change in shape accuracy in squares originated from measurement error. The above proves that SDPM can build the models with a high local shape accuracy of the parts.

## 4. Conclusions

In this work, the scalable DLP printing method was developed to realize the printing of parts with a global large size and local small features. Moreover, the feature has a high accuracy. In the equipment, the range of the effective projection distance was determined at 20.5–30.5 cm through orthogonal tests. Accordingly, the printing formats are 12.35 cm × 6.95 cm and 18.37 cm × 10.34 cm. The smallest pixel feature was printed under the formats, and two types of molding resolutions, 101 μm and 157 μm, were realized on this basis. The shape accuracy contrast test was carried out on the printer, and the samples for NDPM and SDPM were fabricated. The shape accuracy of the small-feature parts was significantly improved. In particular, the average roundness decreased by 49%, and the average straightness of the diamonds declined by 42%.

The building size is influenced by the dimensions of the three linear modules in the mechanical structure and the hardware performance of the projector. In the present work, the length of the three linear modules is 60 cm, and the projection distance is 52 cm. In addition, the short-focus lens can be used to enlarge the building size. The printing accuracy can be enhanced by the error compensation of the mechanism or by using a high-resolution DMD chip.

## Figures and Tables

**Table 1:** Projector parameters.

DMD Model	Wavelength of Light Source	Resolution	Aspect Ratio	Projection Ratio	Contrast
DLP 3010	405 nm	1280 × 720	16:9	1.66	1000:1

**Table 2:** Material properties of photosensitive resin.

Supplier	Viscosity	Density	Wavelength Ranges	Color
Anycubic	150–250 mPa.s	1.15–1.2 g/cm^3^	365–405 nm	White

**Table 3:** Factors level data.

Level	Factors		
	A (mm)	B (s)	C (s)
1	0.08	2.5	6
2	0.1	3	8
3	0.12	3.5	10

**Table 4:** Orthogonal test results.

Number	A (mm)	B (s)	C (s)	ΔX (%)	ΔY (%)	ΔZ (%)
1	0.08	2.5	6	1.21	1.35	2.13
2	0.08	3	8	0.91	1.87	1.72
3	0.08	3.5	10	1.78	2.16	2.38
4	0.1	2.5	8	2.03	1.58	2.93
5	0.1	3	10	1.56	1.62	2.66
6	0.1	3.5	6	2.15	2.87	3.01
7	0.12	2.5	10	1.83	2.43	3.62
8	0.12	3	6	1.22	1.52	2.71
9	0.12	3.5	8	2.37	2.44	3.47

**Table 5:** Range analysis of RSE in Z direction (%).

Data ^1^	A	B	C
K_1_	2.08	2.89	2.62
K_2_	2.87	2.36	2.71
K_3_	3.27	2.95	2.89
R	1.19	0.59	0.27

^1^ K_i_ (i = 1, 2, 3) is the mean at level i.

**Table 6:** Molding formats of large-size and small-feature printing distances.

Projection Distance	Molding Format	Molding Pixel Width
30.5 cm	18.37 cm × 10.34 cm	143.5 μm
20.5 cm	12.35 cm × 6.95 cm	96.5 μm

**Table 7:** Shape accuracy data.

Shape	Accuracy	Method	Number of Tests				
			1	2	3	4	5
Round	Roundness (μm)	NDPM	85	77	86	88	79
		SDPM	43	46	45	38	38
Diamond	Straightness (μm)	NDPM	67	71	65	69	72
		SDPM	39	41	35	38	45
Square	Straightness (μm)	NDPM	23	18	16	19	16
		SDPM	21	14	18	17	20

## Data Availability

The original contributions presented in this study are included in the article.
